# Novel Metformin-Encapsulating Poly(lactic-co-glycolic acid) Microspheres in Calcium Phosphate Pulp-Capping Cement with Dental Pulp Stem Cells for Regenerative Applications

**DOI:** 10.3390/ma19030487

**Published:** 2026-01-26

**Authors:** Mohammad Alenizy, Abdullah Alhussein, Nader Almutairi, Ibrahim Ba-Armah, Heba Alqarni, Yazeed Altamimi, Ayman Altamimi, Tao Ma, Man-Kyo Chung, Michael D. Weir, Abraham Schneider, Hockin H. K. Xu

**Affiliations:** 1 Dental Biomedical Sciences Ph.D. Program, Graduate School, University of Maryland, Baltimore, MD 21201, USA; 2 Department of Biomaterials and Regenerative Dental Medicine, University Maryland School of Dentistry, Baltimore, MD 21201, USA; 3 Department of Restorative Dental Sciences, University of Ha’il, Hail 55475, Saudi Arabia; 4 Department of Restorative Dental Science, College of Dentistry, King Saud University, Riyadh 11545, Saudi Arabia; 5 Department of Conservative Dental Sciences, College of Dentistry, Prince Sattam bin Abdulaziz University, Alkharj 11942, Saudi Arabia; 6 Department of Restorative Dental Sciences, College of Dentistry, Imam Abdulrahman Bin Faisal University, Dammam 31441, Saudi Arabia; 7 Department of Pediatric Dentistry and Orthodontics Sciences, College of Dentistry, King Khalid University, Abha 62521, Saudi Arabia; 8 Department of Oncology and Diagnostic Sciences, School of Dentistry, University of Maryland, Baltimore, MD 21201, USA; 9 Department of Neural and Pain Sciences, School of Dentistry, Center to Advance Chronic Pain Research, University of Maryland, Baltimore, MD 21201, USA; 10 Marlene and Stewart Greenebaum Cancer Center, School of Medicine, University of Maryland, Baltimore, MD 21201, USA; 11 Center for Stem Cell Biology & Regenerative Medicine, School of Medicine, University of Maryland, Baltimore, MD 21201, USA

**Keywords:** metformin, direct pulp capping, biomaterial, tissue regeneration

## Abstract

Metformin is a promising small molecule for dentin regeneration, but an effective local delivery system for pulp applications has been underexplored. This study encapsulated metformin in poly(lactic-co-glycolic acid) (PLGA) microspheres and incorporated them into calcium phosphate–chitosan cement (CPCC) as a direct pulp-capping material (DPC). Metformin-PLGA microspheres were prepared by double emulsion and mixed with CPCC at a concentration of 0% to 20% by weight. Microsphere morphology, encapsulation efficiency, chemical composition, and physico-mechanical properties were characterized, and compatibility with human dental pulp stem cells (hDPSCs) was evaluated by live/dead assay and SEM. The microspheres were spherical (5.43 ± 0.17 µm) with (51 ± 3.69%) encapsulation efficiency, and FTIR confirmed metformin incorporation. The 15% Met-PLGA-CPCC group showed flexural strength (15.22 ± 1.98 MPa), elastic modulus (4.60 ± 0.73 GPa), and work of fracture (104.96 ± 12.48 J/m^2^) comparable to or higher than CPCC and MTA, while all Met-PLGA-CPCC groups had shorter setting times ranging from 18 min to 27 min than CPCC (39.15 ± 2.10 min) and MTA (123 ± 4.2 min). Metformin release increased proportionally with Met-PLGA content. hDPSCs exhibited good attachment and high viability on all materials over the evaluated period. In conclusion, Met-PLGA-CPCC provides fast-setting and favorable physico-mechanical properties, sustained metformin delivery, and excellent hDPSC compatibility. These properties support its potential as a bioactive direct pulp-capping material and as a versatile platform for regenerative applications.

## 1. Introduction

A tooth consists of a highly mineralized outer enamel layer that protects the underlying dentin, a less mineralized collagen-rich tissue [1,2]. Dentin surrounds the dental pulp, a soft connective tissue in the center of the tooth that contains blood vessels, nerves, and diverse cellular composition responsible for dentin formation, tooth nourishment, and sensing external stimuli [3]. Although the dentin–pulp complex has inherent defense mechanisms, pulp tissue may lose its vitality following dental caries, mechanical trauma, or accidental operative procedures that result in direct pulp exposure, allowing microbial irritants to induce inflammatory responses beyond the pulp’s reparative capacity if left untreated. Preserving pulp vitality is therefore crucial to maintaining normal tooth function and proper root development, especially in young patients with immature permanent teeth where the root is not fully formed [4,5]. Moreover, restorative dental treatments have poorer long-term prognosis when performed on non-vital teeth than those performed on vital teeth [6]. A range of regenerative strategies, including bioactive capping materials, growth factor delivery, and stem cell based approaches, has been explored to stimulate dentin–pulp repair and regeneration [7]. Direct pulp capping is a conservative treatment that aims to save pulp vitality by isolating the exposed pulp tissue and promoting reparative dentin formation. Although MTA remains a widely used material for DPC due to its favorable biocompatibility and sealing ability, its clinical performance is hindered by a long setting time, handling challenges, high cost, and the risk of tooth discoloration [8].

Calcium phosphate cement (CPC) has been used in bone repair for more than a century. The development of self-setting CPC in the early 1980s broadened its clinical applications and paved the way for commercial production in the following decade [9]. CPC is effective for repairing bone defects due to its biocompatibility, bioactivity, and ability to be molded into complex defect sites [10]. CPC not only supports new bone formation but also promotes angiogenesis, which is vital for the integration and long-term stability of regenerated tissue [11,12]. Its superior biocompatibility is linked to its rapid conversion to hydroxyapatite (HA), a mineral phase resembling the inorganic component of natural mineralized tissue [13,14]. Among the various CPC formulations, the material used in this study was a calcium phosphate–chitosan cement (CPCC). The powder phase consists of tetracalcium phosphate and dicalcium phosphate anhydrous, which react in an aqueous environment to form hydroxyapatite. Mixed with a chitosan liquid, this system produces a cohesive, moldable paste with enhanced early mechanical strength suitable for small, irregular craniofacial defects [15].

Metformin is an FDA-approved medication that is used to treat patients with type II diabetes mellitus and has a long record of safety. Beyond glycemic control, metformin exhibits anti-inflammatory, antioxidant, and anti-aging properties [16,17]. Systemic administration of metformin may be associated with gastrointestinal side effects and, in rare cases, lactic acidosis [18,19]. Many studies have shown that local delivery of metformin can help recruit stem cells to regenerate mineralized tissues, including bone and dentin, by activating the AMP-activated kinase (AMPK) signaling pathway [20]. There is accumulating evidence that metformin stimulates the expression of genes involved in dentin synthesis: dentin sialophosphoprotein (DSPP), Dentin Matrix Protein 1 (DMP1), Runt-related transcription factor 2 (Runx2), alkaline phosphatase (ALP), and collagen type I alpha 1 chain (COL1A1) [21]. However, a major challenge of hydrophilic drugs like metformin is their rapid release over a short period, as previously reported [22]. Therefore, protecting metformin with a suitable carrier that reduces this rapid release could extend its release and enhance its beneficial effects.

Local drug loading can be achieved by physical methods, such as encapsulation in microspheres or entrapment in a matrix, or by chemical methods that rely on covalent or strong ionic interactions between the drug and the carrier [23,24]. Among the various carrier systems used for physical and chemical drug loading, polymer-based materials are desirable because their composition and structure can be tailored to control degradation and drug release. In particular, poly(lactic-co-glycolic acid) (PLGA) has become one of the most commonly used polymers as a drug carrier due to its excellent biocompatibility, tunable degradation profile, and capacity to encapsulate a broad range of therapeutic agents [25,26].

This study aimed to: (1) encapsulate metformin within PLGA microspheres (Met-PLGA); (2) develop Met-PLGA-CPCC as a pulp capping material with improved physio-mechanical properties compared to CPCC and MTA; (3) measure the release of metformin from the Met-PLGA-CPCC material; and (4) evaluate the effect of Met-PLGA-CPCC on the attachment and proliferation of hDPSCs. The following hypotheses were tested: (1) the incorporation of Met-PLGA microspheres into CPCC would result in physico-mechanical properties comparable to or superior to those of CPCC and MTA; (2) increasing the content of Met-PLGA microspheres within the CPCC matrix would lead to a concentration-dependent increase in metformin release; and (3) Met-PLGA-CPCC would support hDPSC attachment and viability at levels comparable to CPCC alone and MTA.

## 2. Materials and Methods

### 2.1. Preparation of Met-Loaded PLGA

Met-PLGA microspheres were prepared using a modified double emulsion (water in oil in water, W/O/W) solvent diffusion method. A total of 1.5 g of PLGA (50:50, IV 0.6 dL/g; Sigma Aldrich, St. Louis, MO, USA) was dissolved in 10 mL of dichloromethane (DCM, anhydrous, ≥99.8%, stabilized with 150 ppm amylene; Sigma Aldrich, USA) to form the organic phase. Metformin was dissolved at 272 mg/mL in ultrapure water containing 1% (*w*/*v*) polyvinyl alcohol (PVA, Mₙ 13,000–23,000; Sigma Aldrich, CAS 9002 89 5, USA) and 1% (*w*/*v*) NaCl. A 600 µL (containing ~163.2 mg metformin) aliquot of the metformin solution was added to 3 mL of the PLGA/DCM phase and emulsified using a probe sonicator (NBK, Shanghai, China) at 50% amplitude pulse mode equipped with a 3 mm probe in an ice bath for 2 min to form the primary W/O emulsion. This emulsion was then transferred into 6 mL of an external aqueous phase (1% PVA + 1% NaCl) and emulsified again under identical sonication conditions to generate the W/O/W emulsion. The resulting emulsion was poured into 8 mL of ultrapure water containing 1% PVA and 1% NaCl, under magnetic stirring, to facilitate solvent diffusion and particle solidification. The organic solvent was allowed to evaporate overnight under continuous stirring. The microspheres were collected by centrifugation at 2934× *g* for 10 min, washed twice with ultrapure water, and lyophilized for storage.

### 2.2. Preparation of CPCC

CPC powder was composed of tetracalcium phosphate (TTCP; Ca_4_(PO_4_)_2_O) and dicalcium phosphate anhydrous (DCPA; CaHPO_4_). TTCP was synthesized by a solid-state reaction using equimolar amounts of DCPA and calcium carbonate (both from J.T. Baker, Phillipsburg, NJ, USA). The mixture was heated to 1500 °C for 6 h in a furnace (LHTCT, Nabertherm, New Castle, DE, USA), then rapidly cooled to room temperature inside a desiccator. The solid product was ground and sieved to obtain an average particle size of 17 µm. DCPA was separately ground in 95% ethanol for 24 h using a planetary ball mill (PM-100, Retsch, Haan Mettman, Germany), yielding a fine powder with an average particle size of 1 µm. CPC powder was prepared by mixing TTCP and DCPA at a 1:1 molar ratio.

The chitosan malate solution used as the liquid phase in CPCC was prepared by dissolving high-molecular-weight chitosan (800–2000 cps; Sigma-Aldrich, St. Louis, MO, USA) in 60% (*w*/*w*) malic acid (Sigma-Aldrich, St. Louis, MO, USA). The chitosan was then lyophilized for storage. Prior to use, it was reconstituted and used at a 10% chitosan/(chitosan + water) mass fraction in the CPCC formulation.

### 2.3. Characterizations of Met-PLGA Microspheres

The Met-PLGA microspheres were characterized for morphology, sphere size, encapsulation efficiency (EE), and chemical composition.

For scanning electron microscopy (SEM) analysis, dried microspheres were smeared onto glass mounted on metal stubs using double-sided tape, then coated with a 20 nm layer of platinum using a sputter coater under vacuum for 30 s. Surface morphology was then visualized using an SEM (Quanta 200, FEI, Hillsboro, OR, USA).

Particle size distribution was evaluated using a laser diffraction particle size analyzer (Shimadzu SALD-2300, Kyoto, Japan). A total of 1 mL of the Met-PLGA microsphere suspension in ethanol was introduced into the analyzer to measure the size distribution.

The EE from triplicates was determined indirectly by subtracting the amount of metformin remaining in the aqueous phase after centrifugation from the total metformin initially used in the preparation utilizing high-performance liquid chromatography coupled with mass spectrometry (LC-MS/MS), as shown in the following equation:

$$\mathrm{EE}\%=\frac{\mathrm{Total}\;\mathrm{Inital}\;\mathrm{Met}\;\mathrm{Amount}-\mathrm{Free}\;\mathrm{Met}\;\mathrm{in}\;\mathrm{Supernatant}}{\mathrm{Total}\;\mathrm{Met}\;\mathrm{Amount}}\times 100$$

Fourier transform infrared spectroscopy (FTIR) was performed using a Nicolet 6700 spectrophotometer (Thermo Fisher Scientific, Waltham, MA, USA) over 4000–400 cm^−1^ at 4 cm^−1^ resolution using 32 scans per sample (ATR mode) to analyze the chemical structure of the microspheres and verify the presence of metformin within the PLGA.

### 2.4. Mechanical and Physical Properties Evaluation

To evaluate the effect of Met-PLGA concentration on the physical and mechanical properties of CPCC, specimens were divided into five groups, each containing a different percentage of Met-PLGA (0%, 5%, 10%, 15%, and 20% by weight relative to the total powder content). The solid-to-liquid (S/L) ratio was maintained at 3.25:1 across all groups by adjusting the CPC content accordingly. All formulations included 10% chitosan malate as the liquid phase. One extra group was allocated for the inclusion of an MTA commercial product (ProRoot MTA, Dentsply Sirona, Tulsa, OK, USA). The compositions of the experimental groups are presented in Table 1.

#### 2.4.1. Flexural Strength, Elastic Modulus, and Work-of-Fracture

CPCC bars were fabricated using stainless steel molds measuring 4 × 3 × 25 mm. After filling, the molds were placed in a chamber at 37 °C and 100% humidity for 4 h, then demolded. MTA samples for mechanical testing were fabricated by mixing the components following the manufacturer’s instructions. All specimens were then stored in distilled water at 37 °C for 24 h before testing.

Flexural strength, elastic modulus, and work of fracture (n = 6) were evaluated using a three-point bending test with a 20 mm span and a crosshead speed of 1 mm/min on a computer-controlled universal testing machine (MTS Insight 1, Cary, NC, USA). Work of fracture was calculated from the area under the load–displacement curve, normalized to the specimen’s cross-sectional area [17]. The test was terminated at a crosshead displacement of 1 mm.

Flexural strength was calculated using:

$$S=\frac{3\times F_{max}\times L}{2\times b\times h^{2}}$$

where F_max_ is the maximum load at fracture in Newtons; L is the distance in mm between the supports; b is the width of the specimen in mm; h is the height in mm.

Elastic modulus (E) was calculated from the linear region of the load–displacement curve using:

$$E=\left(\frac{F}{C}\right)\times \left(\frac{L^{3}}{\left(4\times b\times h^{2}\right)}\right)$$

where F/C is the slope of the linear elastic portion of the curve.

#### 2.4.2. Flowability and Film Thickness

Samples were prepared and tested according to ISO 6876 [27]. Three specimens (n = 3) were mixed on a glass slab using a stainless-steel spatula until a homogeneous consistency was achieved. Subsequently, 0.5 mL of each material was dispensed onto the center of a polished glass plate measuring 40 × 40 mm. A second glass plate was then placed on top, centered over the material, and a 120 g weight was applied. Ten minutes after mixing, the weight was removed. The maximum and minimum diameters of the compressed material were measured using a digital caliper. Two conditions were required to validate the test: the difference between the maximum and minimum diameters had to be ≤1.0 mm, and the compressed material had to retain a uniform shape.

Film thickness was measured using two glass plates with a contact surface area of 200 ± 10 mm^2^. The combined thickness of the plates was measured with a micrometer. A total of 0.5 mL of the test material was placed at the center of one plate, and the second plate was placed on top. A vertical load of 150 N was applied to the upper plate. Ten minutes after mixing began, the total thickness, including both plates and the material in between, was measured again. Film thickness was calculated by subtracting the initial combined plate thickness from the final measurement, and the results were recorded to the nearest millimeter. The mean value was then determined.

#### 2.4.3. Setting Time

The setting time was measured using the Gilmore needle method (n = 3) in accordance with ASTM C266-03 [28] (ASTM International, West Conshohocken, PA, USA). The final setting time was defined as the duration required for the specimen to resist indentation by the heavier Gilmore needle (453.6 g load, 1.06 mm tip diameter).

#### 2.4.4. Water Sorption, Porosity, and Solubility

This test aimed to evaluate the effect of incorporating Met-PLGA microspheres into CPCC on water sorption, solubility, and porosity. For each group, disk-shaped samples (n = 10, d = 8 mm t = 1 mm) were placed in a vacuum desiccator containing silica desiccant at 37 °C for 24 h to ensure complete evaporation of residual liquid. The samples were then weighed to determine the initial dry mass (m_i_).

Next, the samples were individually immersed in 25 mL of deionized (DI) water and stored at 37 °C for seven days. Following immersion, the samples were reweighed to obtain the saturated mass (m_s_).

To determine the final dry mass, the samples were returned to the vacuum desiccator with silica desiccant and dried again at 37 °C for 24 h. They were then reweighed to obtain the final dry mass (m_f_).

Water sorption, solubility, and porosity were calculated using the following formulas:

Water sorption, expressed as a percentage, was determined by the mass gain after immersion:

$$\mathrm{Water}\;\mathrm{Sorption}\;\left(\%\right)=\frac{m_{s}-m_{i}}{m_{i}}\times 100$$

Water solubility was determined by the mass loss after immersion:

$$\mathrm{Water}\;\mathrm{Solubility}\;\left(\%\right)=\frac{m_{f}-m_{i}}{m_{i}}\times 100$$

Porosity was calculated using the fluid displacement method, where V_p_ is the pore volume:

$$V_{p}=\frac{m_{s}-m_{f}}{\mathrm{Water}\;\mathrm{density}}$$

$$\mathrm{Porosity}\;\left(\%\right)=\frac{V_{p}}{\mathrm{sample}\;\mathrm{volume}}\times 100$$

### 2.5. Metformin Release

Metformin release from disks (d = 8 mm, t = 1 mm; n = 4) was evaluated by placing disks from each group in airtight vials containing 1 mL of deionized water. Disks were transferred to fresh vials every 24 h from day 1 to day 7, and subsequently every seven days until day 28. Sink conditions were maintained throughout the release study by complete replacement of the release medium at each sampling time point. Aliquots collected on days 1, 2, 3, 4, 5, 6, 7, 14, 21, and 28 were analyzed for metformin release using LC-MS/MS [29]. LC-MS/MS analysis was conducted on a TSQ Altis Triple Stage Quadrupole Mass Spectrometer coupled with an Ultimate 3000 RS Liquid Chromatography system (Thermo Scientific, San Jose, CA, USA). Data acquisition and analysis were performed using Xcalibur V 2.1 (Thermo Scientific, San Jose, CA, USA). The mass spectrometer operated in positive ion mode, monitoring m/z 130.0 → 60.0 for metformin and m/z 136.0 → 60.1 for metformin_d6 (internal standard), using an electrospray ionization (ESI) source. Metformin quantification was performed within a concentration range of 5–5000 ng/mL. Samples were diluted with water and acetonitrile (50:50, *v*/*v*) and chromatographed on a Zorbax SB C18 column (4.6 × 100 mm, 3.5 µm; Agilent Technologies, Santa Clara, CA, USA) using an isocratic mobile phase of 0.1% formic acid in water and acetonitrile (30:70, *v*/*v*) at a flow rate of 0.6 mL/min.

### 2.6. Viability and Proliferation Assays of hDPSCs

Cell attachment and proliferation were assessed using a live/dead staining assay to evaluate the impact of incorporating Met-PLGA on hDPSCs. Disks were seeded with hDPSCs in 48-well plates at a density of 2.5 × 10^4^ cells per well in 1 mL of basal medium (Lonza, MD, USA) supplemented with 10% mesenchymal cell growth supplement (MCGS), 2% L-glutamine, 1% ascorbic acid, and 0.1% gentamicin sulfate-amphotericin B (GA).

At days 1, 4, and 7, samples were rinsed with PBS and incubated at 37 °C for 30 min with a live/dead staining solution (Invitrogen, Carlsbad, CA, USA) containing 4 µM calcein AM and 2 µM ethidium homodimer-1. Live cells exhibited green fluorescence due to intracellular enzymatic conversion of calcein AM, while dead cells showed red fluorescence as ethidium homodimer-1 binds to DNA in cells with compromised membranes.

Fluorescence imaging was performed on a Cytation 5 imaging system (Biotek, Santa Clara, CA, USA). Three random images per disk were used, yielding nine images per group at each time point (n = 3). Live cell density and viability were quantified from the fluorescence images using ImageJ (version 1.54g). The percentage of viable cells (VC%) was then calculated as: VC% = [live cells/(live cells + dead cells)] × 100.

### 2.7. SEM

SEM (Apreo 2 S, Thermo Fisher Scientific, Waltham, MA, USA) was used to examine the surface and internal morphology of the disks, as well as hDPSC attachment on the CPCC and Met-PLGA-CPCC samples after 7 days from cell seeding. Samples were rinsed with PBS and fixed in 1% glutaraldehyde at 4 °C overnight. Then, they were rinsed again with PBS and dehydrated through a graded ethanol concentration. After dehydration, specimens were treated with hexamethyldisilazane and left to air-dry overnight. Finally, the dried samples were sputter-coated with platinum and viewed under SEM.

### 2.8. Statistical Analysis

Statistical analyses were performed using SigmaPlot software (version 12.0; SYSTAT, Chicago, IL, USA). Data normality was determined using the Shapiro–Wilk test. One-way analysis of variance (ANOVA) followed by Tukey’s post hoc test was used to evaluate differences among groups. A *p*-value < 0.05 was considered statistically significant.

## 3. Results

### 3.1. Characterizations of Met-PLGA Microspheres

**Figure 1:**
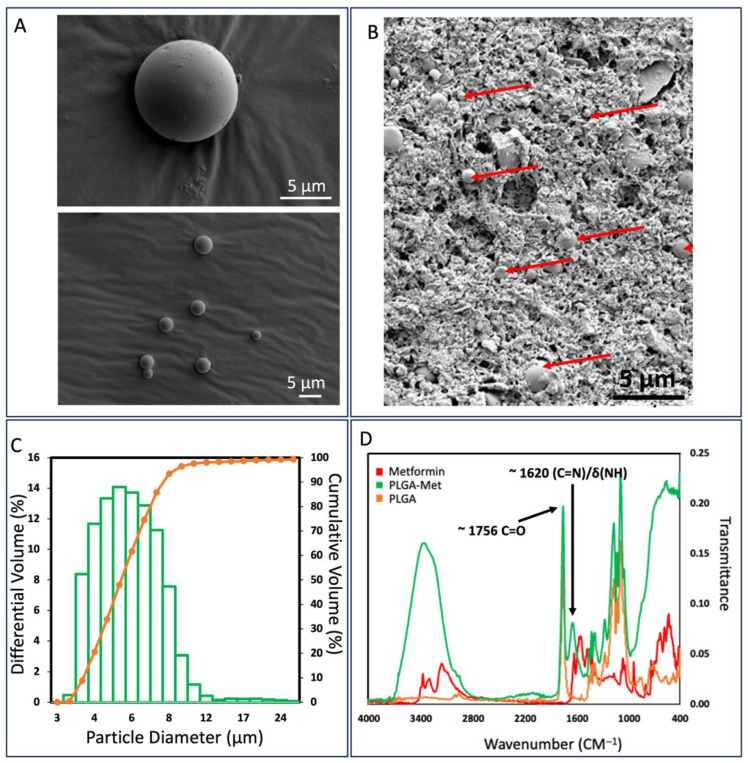
(**A**) SEM micrographs of Met-PLGA microparticles. The particles are predominantly spherical with smooth, continuous shells and minimal surface pitting. No wrinkling or shell collapse was observed, consistent with adequate polymer solidification during the emulsion–solvent-evaporation process. (**B**) SEM image of a 20% Met-PLGA-CPCC disk. The uniform morphology and limited agglomeration of Met-PLGA microparticles enabled uniform dispersion within the CPCC matrix (red arrows). (**C**) Laser-diffraction particle sizing (Shimadzu SALD-2300) showed a unimodal, volume-based distribution centered at ~5–6 µm, with most particles between ~4–9 µm and cumulative volume reaching 100% by 10 µm. (**D**) FTIR spectra of PLGA, metformin, and Met-PLGA. Met-PLGA retained PLGA peaks and displayed metformin bands with slight broadening/shifts (~3–10 cm^−1^), confirming metformin incorporation.

Figure 1A,B shows the SEM micrograph of Met-PLGA and the 20% Met-PLGA-CPCC disk surface. The microspheres were predominantly spherical with smooth, continuous shells, and minimal surface pitting. No wrinkling or shell collapse was observed, consistent with adequate polymer solidification during the emulsion–solvent-evaporation process. The regular morphology and limited agglomeration are suitable for incorporation into CPC; these intact spheres are dispersed uniformly within the cement matrix and provide reproducible interfacial contact, supporting controlled metformin release (Figure 1B).

Met-PLGA microspheres had a mean diameter of (5.43 ± 0.17) µm and an encapsulation efficiency of (51.02 ± 3.69)% (Table 2). Laser-diffraction sizing (Shimadzu SALD-2300) showed a unimodal, volume-based distribution centered at ~5–6 µm, with most microspheres between ~4 and 9 µm and the cumulative volume approaching 100% by ~10 µm (Figure 1C).

Figure 1D presents the FTIR spectra of PLGA, metformin, and Met-PLGA. Neat PLGA showed an ester carbonyl stretch at 1755–1758 cm^−1^, a C–O–C stretching envelope at 1185–1080 cm^−1^, and aliphatic C–H modes at 2990–2940 cm^−1^. Neat metformin exhibited prominent bands at 1665–1595 cm^−1^ (overlapping ν(C=N)/δ(NH)), additional δ(NH)/ν(C–N) features at 1575–1510 cm^−1^, and complex C–N/C–N^+^ vibrations between 1250–1020 cm^−1^. The Met-PLGA spectrum retained the PLGA carbonyl near ~1756 cm^−1^ and the C–O–C band set while displaying metformin-associated bands at ~1655–1605 and ~1570–1510 cm^−1^ with modest broadening and small shifts (≈3–10 cm^−1^). The defining peaks of PLGA and metformin, along with these subtle shifts and band broadening, support the presence of metformin within the Met-PLGA microspheres [30,31].

### 3.2. Flexural Strength, Elastic Modulus, and Work of Fracture

**Figure 2:**
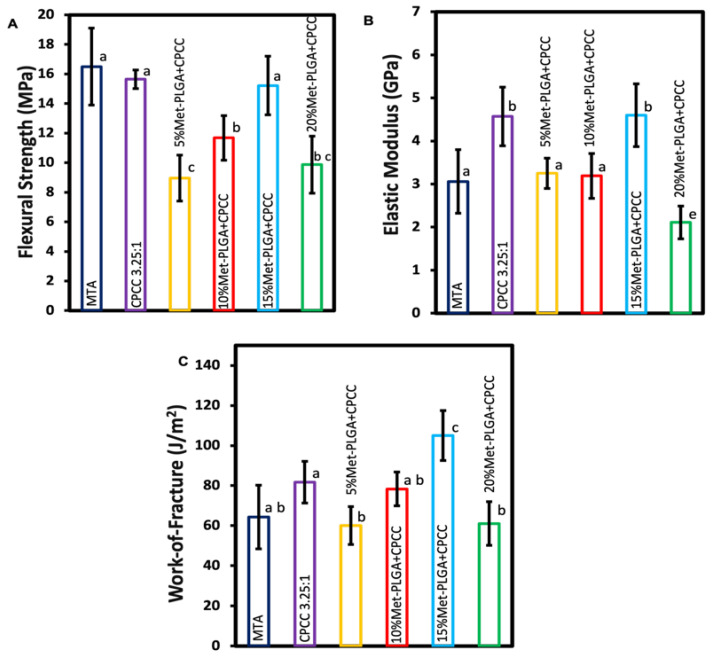
(**A**) Flexural strength, (**B**) elastic modulus, and (**C**) work of fracture of various Met-PLGA-CPCC content compared to CPCC and MTA. Met-PLGA-CPCC at 15% loading showed comparable or higher value to CPCC and MTA. Values with different letters indicate significant differences (*p* < 0.05).

Flexural strength, elastic modulus, and work of fracture are presented in Figure 2 (mean ± SD; n = 6). CPCC (15.65 ± 0.63 MPa) and 15% Met-PLGA-CPCC (15.22 ± 1.98 MPa) had comparable flexural strengths to MTA (16.5 ± 2.6 MPa) (*p* > 0.05). The 5% Met-PLGA-CPCC (8.96 ± 1.56 MPa) showed the lowest strength and was significantly lower than all other groups (*p* < 0.05), except the 20% Met-PLGA-CPCC (9.87 ± 1.93 MPa), for which the difference was not significant (*p* > 0.05).

CPCC and 15% Met-PLGA-CPCC demonstrated the highest elastic modulus values at 4.57 ± 0.68 GPa and 4.6 ± 0.73 GPa, respectively, compared to all groups (*p* < 0.05). The 20% Met-PLGA-CPCC (2.11 ± 0.38 GPa) showed a significantly lower elastic modulus than all other groups (*p* < 0.05), and there was no significant difference between the 5% Met-PLGA-CPCC (3.25 ± 0.35 GPa), 10% Met-PLGA-CPCC (3.19 ± 0.52 GPa), and MTA (3.06 ± 0.74 GPa) (*p* > 0.05).

The work of fracture values showed that the 15% Met-PLGA-CPCC (104.96 ± 12.48 J/m^2^) was statistically higher than the other groups (*p* < 0.05). There was no significant difference between CPCC, 10% Met-PLGA–CPCC, and MTA (81.73 ± 10.44 J/m^2^), (78.31 ± 8.43 J/m^2^), and (64.33 ± 15.9 J/m^2^), respectively, (*p* > 0.05).

### 3.3. Flowability and Film Thickness

**Figure 3:**
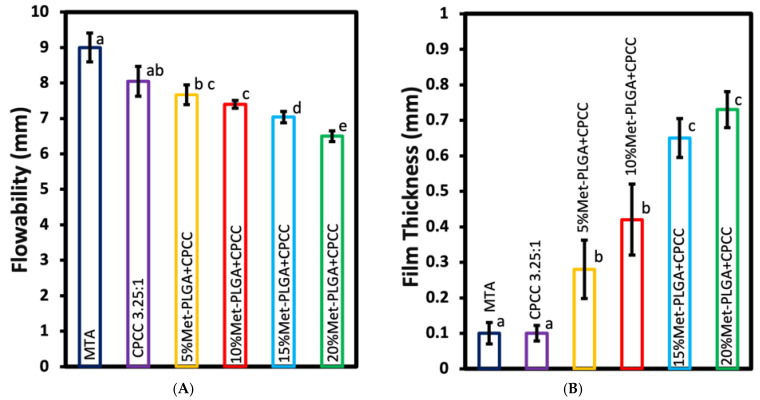
(**A**) Flowability, and (**B**) film thickness of Met-PLGA-CPCC at various concentrations compared to CPCC and MTA. Values with different letters indicate significant differences (*p* < 0.05).

Flowability results are shown in Figure 3A. MTA (9.0 ± 0.41 mm) and CPCC (8.05 ± 0.42 mm) exhibited the highest flowability among all groups (*p* < 0.05). Among the Met-PLGA-CPCC groups, flowability decreased with increasing Met-PLGA content. The 5% Met-PLGA-CPCC showed the highest flowability (7.67 ± 0.28 mm), whereas the 20% Met-PLGA-CPCC showed the lowest (6.5 ± 0.15 mm) (*p* < 0.05). No significant differences were detected between CPCC and 5%, 5% and 10%, or 10% and 15% Met-PLGA-CPCC (*p* > 0.05).

Film thickness values are shown in Figure 3B. MTA (0.1 ± 0.03 mm) and CPCC (0.1 ± 0.02 mm) were associated with significantly thinner film compared to all groups (*p* < 0.05). A general trend of increasing thickness with increasing Met-PLGA content was observed. The 5% (0.28 ± 0.08 mm) and 10% (0.42 ± 0.10 mm) Met-PLGA-CPCC groups had greater thickness than CPCC and MTA, but remained significantly thinner than the 15% (0.65 ± 0.05 mm) and 20% (0.73 ± 0.05 mm) groups (*p* < 0.05).

### 3.4. Setting Time

**Figure 4:**
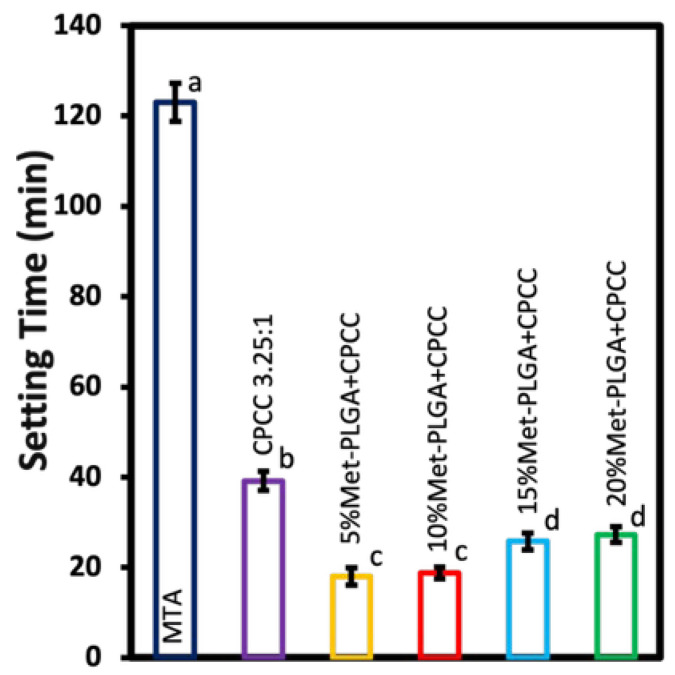
Setting time of different Met-PLGA-CPCC formulations compared to CPCC and MTA. All Met-PLGA-CPCC groups exhibited significantly shorter setting times compared to CPCC and MTA. Values with different letters indicate significant differences (*p* < 0.05).

The setting time results are summarized in Figure 4. CPCC and MTA set at (39.15 ± 2.10 min) and (123.00 ± 4.20 min), respectively. In contrast, all Met-PLGA-CPCC groups showed significantly shorter setting times (*p* < 0.05). 5% Met-PLGA-CPCC and 10% Met-PLGA-CPCC had the shortest setting times compared to all other groups at 18 ± 1.54 min and 18.45 ± 1.15 min, respectively (*p* < 0.05). The setting times for 15% Met-PLGA-CPCC and 20% Met-PLGA-CPCC were significantly shorter than the CPCC but longer than 5% and 10% Met-PLGA-CPCC, at 25.45 ± 1.26 min and 27.15 ± 1.71 min, respectively (*p* < 0.05).

### 3.5. Water Sorption, Solubility and Porosity

**Figure 5:**
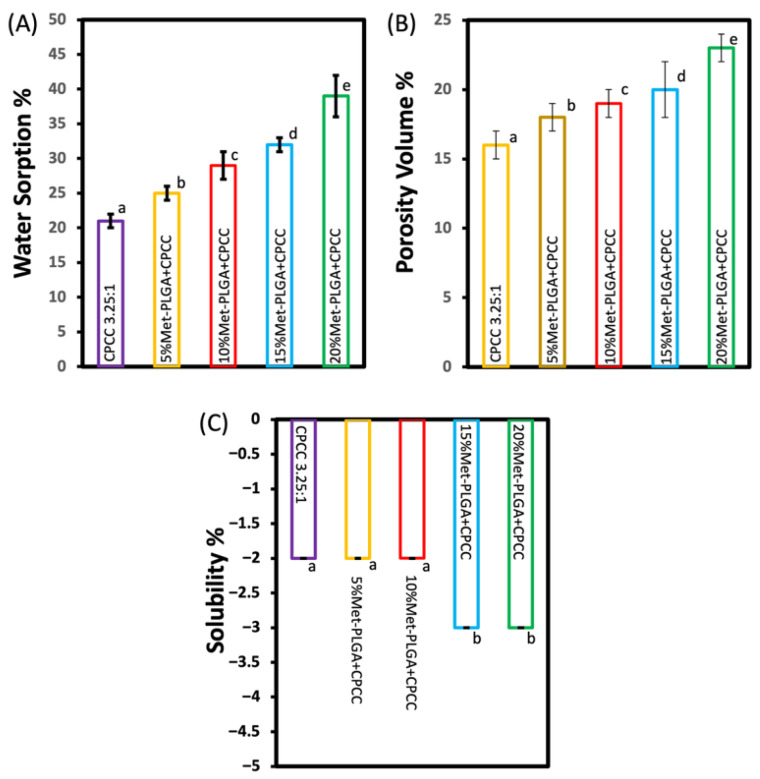
(**A**) Water sorption, (**B**) porosity volume, and (**C**) solubility. The water sorption, porosity, and solubility increase with Met-PLGA percentage increase. Values with different letters indicate significant differences (*p* < 0.05).

The addition of Met-PLGA microspheres to the CPCC significantly affected all three measured properties, as summarized in Figure 5.

An apparent dose-dependent increase in water sorption was observed. The lowest mean value was recorded for the CPCC group (21 ± 1 wt%), which progressively increased in 5% Met-PLGA-CPCC (25 ± 1 wt%), 10% Met-PLGA-CPCC (29 ± 2 wt%), and 15% Met-PLGA-CPCC (32 ± 1 wt%). The maximum value was observed by the 20% Met-PLGA-CPCC group (39 ± 3 wt%), indicating a strong positive correlation between Met-PLGA content and water sorption.

Water porosity also exhibited a significant ascending trend across the formulations. The mean values ranged from the lowest in CPCC (16 ± 1 v%) to the highest in 20% Met-PLGA-CPCC (23 ± 1 v%). Statistical analysis confirmed that each group differed significantly from the others’ means (*p* < 0.05).

Water solubility results confirmed that the specimens lost mass in all groups. However, the addition of more Met-PLGA microspheres (15% and 20%) induced more mass loss within one week compared to the CPCC, 5%, and 10% Met-PLGA-CPCC (*p* < 0.05).

### 3.6. Metformin Release

**Figure 6:**
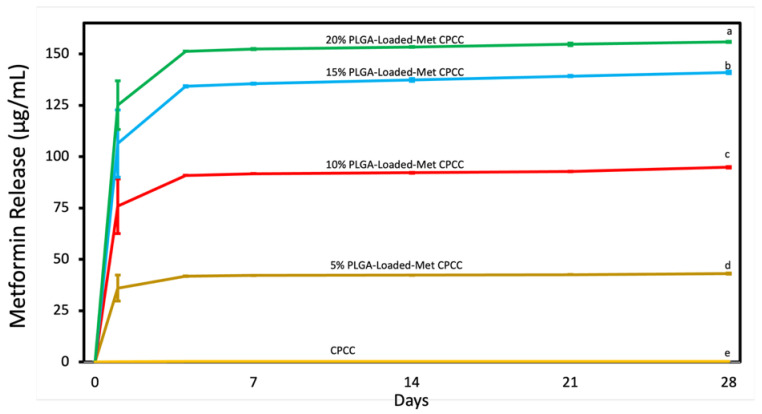
Measurement of released metformin from Met-PLGA-CPCC disks. The amount of released metformin corresponds proportionally to its concentration. Values with different letters indicate significant differences (*p* < 0.05).

Metformin release data are illustrated in Figure 6. The release of metformin was directly proportional to the Met-PLGA concentration in CPCC. At 28 d, 20% Met-PLGA-CPCC released 155.9 ± 0.3 µg/mL, 15% Met-PLGA-CPCC at 141 ± 0.6 µg/mL, 10% Met-PLGA-CPCC at 94.8 ± 0.37 µg/mL, 5% Met-PLGA-CPCC at 43 ± 0.3 µg/mL, and CPCC exhibited no release (*p* < 0.05).

### 3.7. Viability and Proliferation Assays of hDPSCs

**Figure 7:**
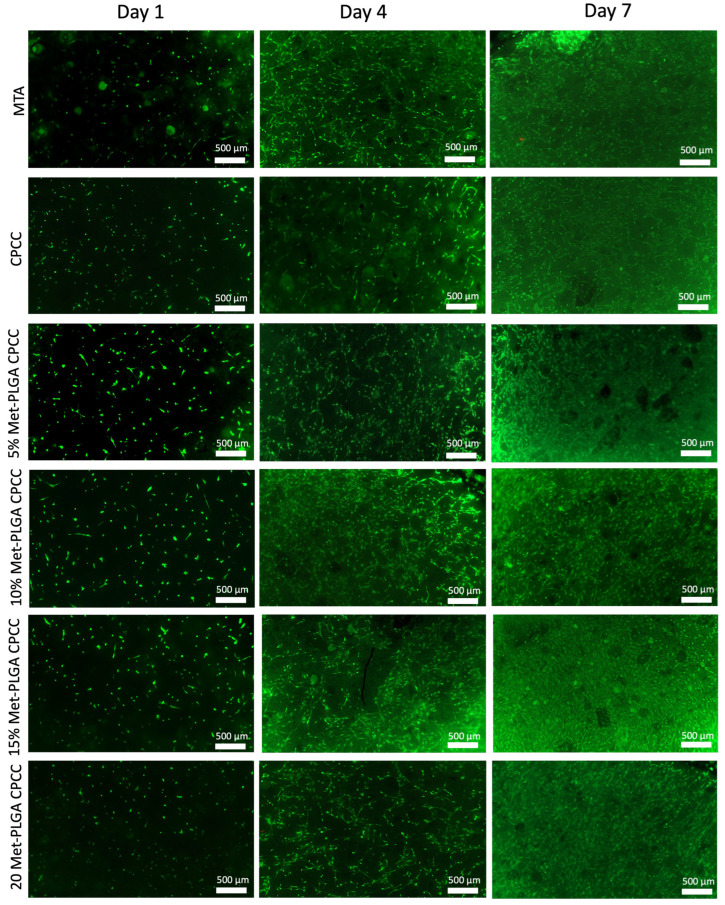
Representative live/dead fluorescence images of hDPSCs on MTA, CPCC, and Met-PLGA-CPCC. Live cells appear green and dead cells red. hDPSCs showed excellent viability on Met-PLGA-CPCC, and comparable to CPCC and MTA.

Figure 7 displays representative live/dead fluorescence images of hDPSCs cultured on the surfaces of MTA, CPCC, 5%, 10%, 15%, and 20% Met-PLGA-CPCC at days 1, 4, and 7. Green fluorescence indicates viable cells, which were distributed uniformly across all groups, suggesting effective cell attachment. Dead cells stained red were scarce, indicating that all materials supported high cell viability and showed excellent biocompatibility.

**Figure 8:**
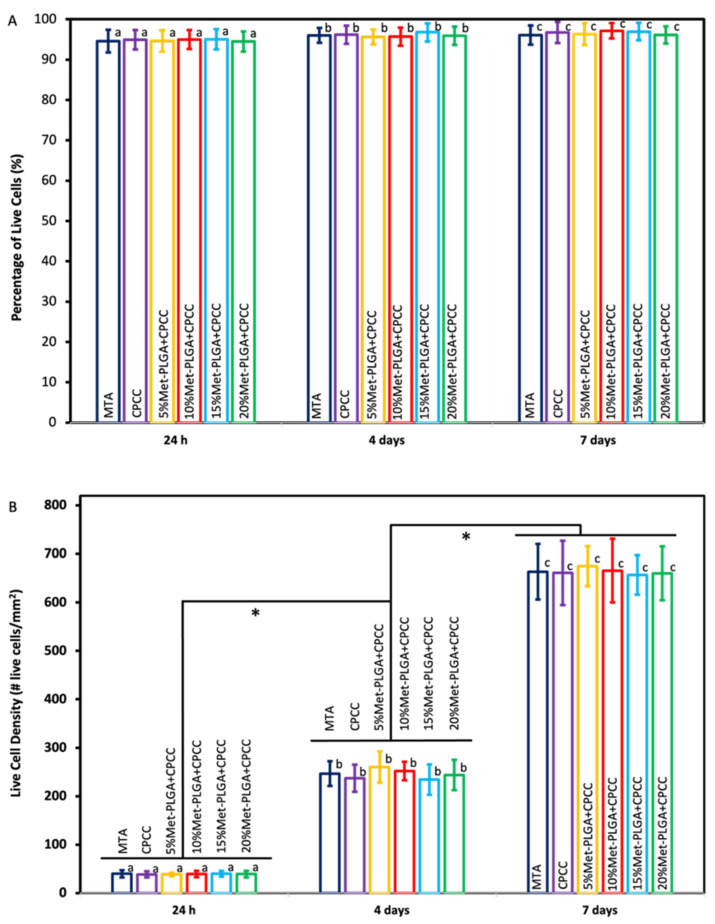
(**A**) Percentage of live cells and (**B**) live cell density per mm^2^ at 1 d, 4 d, and 7 d. Values with different letters indicate significant differences, and * denotes significance difference between time points in (**B**).

Figure 8A,B illustrates the percentage and density of live hDPSCs at 1, 4, and 7 days. There was a significant increase in live cell density over time, reflecting increased cell proliferation (*p* < 0.05). However, no significant differences were found between the groups in either cell viability or density at the same time point (*p* > 0.05). The proportion of viable cells remained high across all groups throughout the 7 days.

### 3.8. SEM

**Figure 9:**
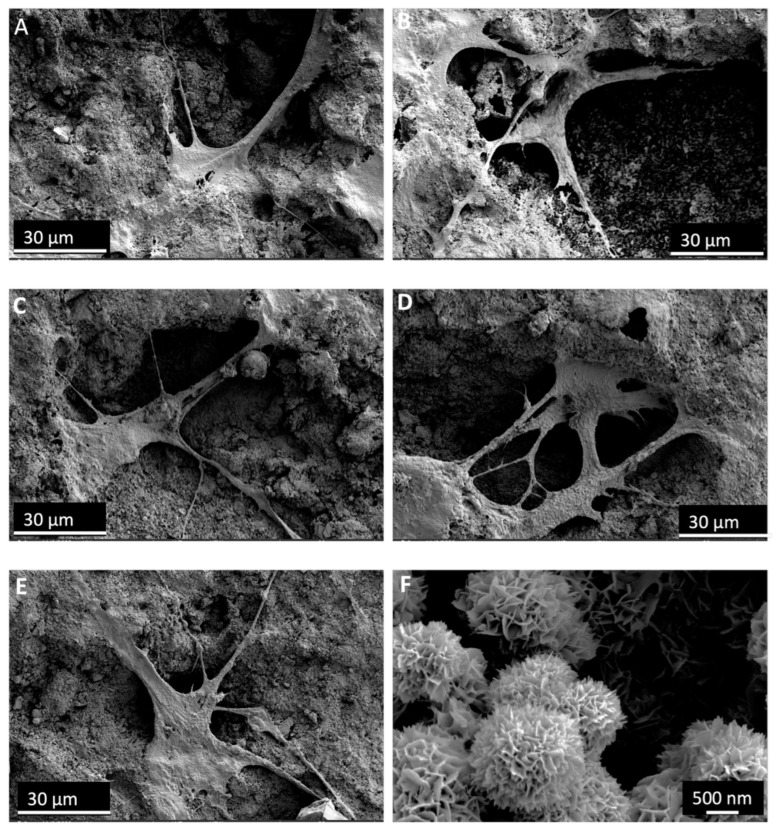
hDPSCs on CPCC and Met-PLGA-CPCC disks at 7 d. (**A**) CPCC, (**B**) 5% Met-PLGA-CPCC, (**C**) 10% Met-PLGA-CPCC, (**D**) 15% Met-PLGA-CPCC, and (**E**) 20% Met-PLGA-CPCC. (**F**) SEM images of high magnification on Met-PLGA-CPCC disk showing flake-like apatite layer, indicating successful cement setting and mineral phase development.

Figure 9F displays SEM images of the CPCC, showing spherical agglomerates with a rosette-like shape made up of radiating needle- and plate-like crystals. This appearance resembles HA formation and indicates complete cement setting and mineral phase development.

Figure 9A–E shows the attachment and morphology of hDPSCs on the surface of the disks after 7 days. hDPSCs demonstrated strong attachment to the material surface, with extended pseudopodia and a spread morphology. The cells interacted closely with the underlying substrate, anchoring to its porous microstructure. Numerous cellular extensions were visible, indicating active adhesion and growth. These results support the material’s favorable surface features for cell attachment and biocompatibility.

## 4. Discussion

This study successfully encapsulated metformin within PLGA microspheres and incorporated them into self-setting CPCC for the first time. The 15% Met-PLGA-CPCC group exhibited improved physico-mechanical properties, with approximately a fivefold reduction in setting time compared to MTA. Metformin was released from the material into the medium, and the release was prolonged compared with metformin incorporated directly into the liquid chitosan [32]. Excellent attachment and proliferation of hDPSCs were observed in all experimental groups by live/dead staining and SEM imaging, indicating that the incorporation of metformin and PLGA did not compromise the CPCC’s excellent biocompatibility.

The double-emulsion technique was employed to encapsulate the hydrophilic metformin within a hydrophobic carrier [33]. The smooth uniform morphology and high encapsulation efficiency of the Met-PLGA microspheres were attributed to the addition of NaCl and PVA [34]. NaCl acts as an osmotic agent, helping balance the osmotic pressure between the organic and aqueous phases. This limits water influx into the droplets, reducing surface pores and producing smoother, more uniform microspheres [35]. PVA serves as an emulsifier and stabilizer at the oil–water interface; at an appropriate concentration, it keeps the emulsion droplets stable and reduces drug loss into the surrounding solution, which improves encapsulation efficiency [36]. The early increase in mass loss is primarily attributed to the degradation of the PLGA microspheres and the release of encapsulated drug, as evidenced by the clear dose-dependent effect. This controlled degradation is essential for creating pathways for the release of the encapsulated metformin [37,38]. Although the mass loss was statistically significant, the absolute solubility values of the experimental materials (approximately 2–3%) and MTA (approximately 1%) remained within the limits specified by ISO 6876-2:2025 [27] and are therefore unlikely to be clinically relevant [39,40].

In a composite where a softer PLGA phase is embedded in a brittle CPC phase, there is an optimal PLGA content at which the mechanical performance is maximized [41]. At low PLGA content (5%), the microspheres mainly behave as defects in the continuous CPC matrix. They disrupt the ceramic network and act as stress concentrators, resulting in lower flexural strength and modulus than CPCC and MTA [42]. When PLGA is increased to an intermediate level (15%), the polymer becomes more uniformly distributed and better bonded, allowing it to bridge and deflect microcracks and dissipate energy through plastic deformation. This improves the work of fracture and provides strength, enabling recovery to values comparable to those of CPCC and MTA. At higher PLGA loading (20%), the polymer phase becomes the dominant phase and is not completely surrounded by CPC, leading to a decrease in strength again [43]. While these trends are consistent with the reported behavior of particulate-reinforced cements, direct fracture surface analysis was not performed for confirmation. However, the mechanical properties of a DPC material should not be the primary determining factor when choosing a DPC material for two reasons: (1) it is applied only as a small, pinpoint layer over the exposure site, and (2) a protective base or definitive restoration is routinely placed on top of the pulp-capping material to shield it from functional loading [44,45]. Nevertheless, the values obtained for flexural strength, elastic modulus, and work of fracture in the Met-PLGA-CPCC group were comparable to those previously reported for other DPC materials [46,47].

Introducing PLGA microspheres into CPCC significantly reduced the setting time compared to CPCC and MTA. This reduction can be attributed to a combination of chemical and rheological effects. From a chemical perspective, the formation of calcium lactate, which occurs when lactic acid groups from hydrolyzed polylactic acid react with calcium ions released from the CPC powder, potentially accelerates the setting reaction [48]. From a rheological perspective, incorporating PLGA into the chitosan liquid increases paste viscosity, which may enhance contact between the CPC powder and the liquid phase and facilitate more efficient cement formation [49]. As the PLGA content increases further, the setting time rises slightly, possibly because the CPC:chitosan ratio decreases as PLGA is added at the expense of CPC powder, which may slow the overall cement formation rate; however, the setting time remains significantly shorter than that of CPCC and MTA [32]. The extended setting time often prevents placement of the definitive restoration in the same visit, necessitating a temporary restoration and an additional appointment. At the subsequent visit, the clinician must re-administer local anesthesia, remove the temporary restoration, and then place the final restoration, thereby increasing chair time and resource utilization [50].

Metformin’s hydrophilicity makes it highly soluble, limiting cellular uptake and posing a challenge for drug encapsulation [51]. Encapsulating metformin within a physical barrier extends its release rate compared to our previous non-encapsulated formulation, thereby enhancing local bioavailability and cellular absorption, leading to greater cell differentiation and dentin regeneration [32,52,53,54]. While the release profile demonstrated an initial phase followed by extended release, detailed kinetic modeling to distinguish diffusion- versus degradation-controlled mechanisms was not performed and represents a limitation of the present study. PLGA degradation is primarily governed by its composition, crystallinity, and molecular weight, with the rate increasing as the glycolic acid content rises, crystallinity decreases, and molecular weight decreases [55]. In addition, unlike MTA, CPC sets via a non-exothermic reaction, so there is no risk of heat generation during the setting reaction; thus, the risk of heat-induced degradation of the active drug is minimal [12,56].

The introduction of PLGA and metformin into CPCC did not prevent HA formation or compromise its excellent cellular biocompatibility, as evidenced by the live/dead assays and SEM. Metformin, PLGA, chitosan, and CPC are all FDA-approved for use in humans: metformin as an oral medication for type II diabetes, PLGA as a biodegradable carrier in various drug-delivery systems and vaccines, chitosan for wound dressings, and CPC for the repair of non-load-bearing bone defects, including defects in the craniofacial region [57,58,59,60].

This study successfully encapsulated metformin within uniform PLGA microspheres, enabling extended drug release without compromising biocompatibility or mechanical properties at a 15% loading. The added Met-PLGA enhances cement biodegradation, extends metformin release, and may improve material porosity and osteoconductivity without compromising the physico-mechanical properties. In addition to its potential as a direct pulp-capping material, these features indicate that Met-PLGA-CPCC could also be used as a moldable scaffold for craniofacial bone regeneration and for other applications where a bioactive cement is needed to provide local, extended metformin delivery.

This study has several limitations. The biological evaluation was limited to in vitro cytocompatibility and short-term hDPSC attachment and viability, without assessment of odontogenic differentiation, mineralized matrix formation, or in vivo validation; therefore, regenerative efficacy was not directly evaluated. Metformin release was examined under simplified in vitro conditions, and qualitative clinical handling properties such as washout resistance and injectability were not assessed. Future in vivo and long-term studies are warranted to confirm its efficacy in promoting dentin formation and preserving pulp vitality.

## 5. Conclusions

The novel Met-PLGA-CPCC exhibited an extended metformin release and possessed excellent physical and mechanical properties, especially with the 15% Met-PLGA-CPCC composition. In addition, Met-PLGA-CPCC had excellent attachment and proliferation of hDPSCs. These findings indicate that Met-PLGA-CPCC is a promising bioactive material for direct pulp-capping applications.

## Figures and Tables

**Table 1:** Experimental groups and material compositions.

Group	Met-PLGA (Parts)	CPC (Parts)	Chitosan (Parts)	S/L Ratio
CPCC (experimental control)	0	3.25	1	3.25:1
5% Met-PLGA-CPCC	0.16	3.09	1	3.25:1
10% Met-PLGA-CPCC	0.32	2.92	1	3.25:1
15% Met-PLGA-CPCC	0.48	2.76	1	3.25:1
20% Met-PLGA-CPCC	0.65	2.60	1	3.25:1
MTA (commercial control)	-	-	-	As per the manufacturer

**Table 2:** Met-PLGA microsphere size and EE.

Met-PLGA Microspheres	
Microsphere Mean Size (µm)	5.42 (0.16)
EE%	51.02 (3.69)

## Data Availability

The original contributions presented in this study are included in the article. Further inquiries can be directed to the corresponding authors.
